# Autophagy is a regulator of TGF-*β*_1_-induced fibrogenesis in primary human atrial myofibroblasts

**DOI:** 10.1038/cddis.2015.36

**Published:** 2015-03-19

**Authors:** S Ghavami, R H Cunnington, S Gupta, B Yeganeh, K L Filomeno, D H Freed, S Chen, T Klonisch, A J Halayko, E Ambrose, R Singal, I M C Dixon

**Affiliations:** 1 Department of Physiology, Manitoba Institute of Child Health, Winnipeg, Manitoba, Canada; 2 Biology of Breathing Group, Manitoba Institute of Child Health, Winnipeg, Manitoba, Canada; 3 Department of Physiology and Institute of Cardiovascular Sciences, St. Boniface Research Centre, University of Manitoba, Winnipeg, Manitoba, Canada; 4 Department of Human Anatomy and Cell Science, University of Manitoba, Winnipeg, Manitoba, Canada; 5 Department of Internal Medicine, Manitoba Institute of Child Health, Winnipeg, Manitoba, Canada; 6 Cardiac Sciences Program, St. Boniface General Hospital, Winnipeg, Manitoba, Canada

## Abstract

Transforming growth factor-*β*_1_ (TGF-*β*_1_) is an important regulator of fibrogenesis in heart disease. In many other cellular systems, TGF-*β*_1_ may also induce autophagy, but a link between its fibrogenic and autophagic effects is unknown. Thus we tested whether or not TGF-*β*_1_-induced autophagy has a regulatory function on fibrosis in human atrial myofibroblasts (hATMyofbs). Primary hATMyofbs were treated with TGF-*β*_1_ to assess for fibrogenic and autophagic responses. Using immunoblotting, immunofluorescence and transmission electron microscopic analyses, we found that TGF-*β*_1_ promoted collagen type I*α*2 and fibronectin synthesis in hATMyofbs and that this was paralleled by an increase in autophagic activation in these cells. Pharmacological inhibition of autophagy by bafilomycin-A1 and 3-methyladenine decreased the fibrotic response in hATMyofb cells. ATG7 knockdown in hATMyofbs and ATG5 knockout (mouse embryonic fibroblast) fibroblasts decreased the fibrotic effect of TGF-*β*_1_ in experimental *versus* control cells. Furthermore, using a coronary artery ligation model of myocardial infarction in rats, we observed increases in the levels of protein markers of fibrosis, autophagy and Smad2 phosphorylation in whole scar tissue lysates. Immunohistochemistry for LC3*β* indicated the localization of punctate LC3*β* with vimentin (a mesenchymal-derived cell marker), ED-A fibronectin and phosphorylated Smad2. These results support the hypothesis that TGF-*β*_1_-induced autophagy is required for the fibrogenic response in hATMyofbs.

Interstitial fibrosis is common to many cardiovascular disease etiologies including myocardial infarction (MI),^1^ diabetic cardiomyopathy^2^ and hypertension.^3^ Fibrosis may arise due to maladaptive cardiac remodeling following injury and is a complex process resulting from activation of signaling pathways, such as TGF-*β*_1_.^4^ TGF-*β*_1_ signaling has broad-ranging effects that may affect cell growth, differentiation and the production of extracellular matrix (ECM) proteins.^5, 6^ Elevated TGF-*β*_1_ is observed in post-MI rat heart^7^ and is associated with fibroblast-to-myofibroblast phenoconversion and concomitant activation of canonical Smad signaling.^8^ The result is a proliferation of myofibroblasts, which then leads to inappropriate deposition of fibrillar collagens, impaired cardiac function and, ultimately, heart failure.^9, 10^

Autophagy is necessary for cellular homeostasis and is involved in organelle and protein turnover.^11, 12, 13, 14^ Autophagy aids in cell survival by providing primary materials, for example, amino acids and fatty acids for anabolic pathways during starvation conditions.^15, 16^ Alternatively, autophagy may be associated with apoptosis through autodigestive cellular processes, cellular infection with pathogens or extracellular stimuli.^17, 18, 19, 20^ The overall control of cardiac fibrosis is likely due to the complex functioning of an array of regulatory factors, but to date, there is little evidence linking autophagy with fibrogenesis in cardiac tissue.^11, 12, 13, 14, 15, 16, 17, 18, 21, 22^

Recent studies have demonstrated that TGF-*β*_1_ may not only promote autophagy in mouse fibroblasts and human tubular epithelial kidney cells^15, 23, 24^ but can also inhibit this process in fibroblasts extracted from human patients with idiopathic pulmonary fibrosis.^25^ Moreover, it has recently been reported that autophagy can negatively^15^ and positively^25, 26, 27^ regulate the fibrotic process in different model cell systems. In this study, we have explored the putative link between autophagy and TGF-*β*_1_-induced fibrogenesis in human atrial myofibroblasts (hATMyofbs) and in a model of MI rat heart.

## Results

### TGF-*β*_1_ simultaneously induces fibrosis and autophagy in hATMyofbs

**Figure 1:**
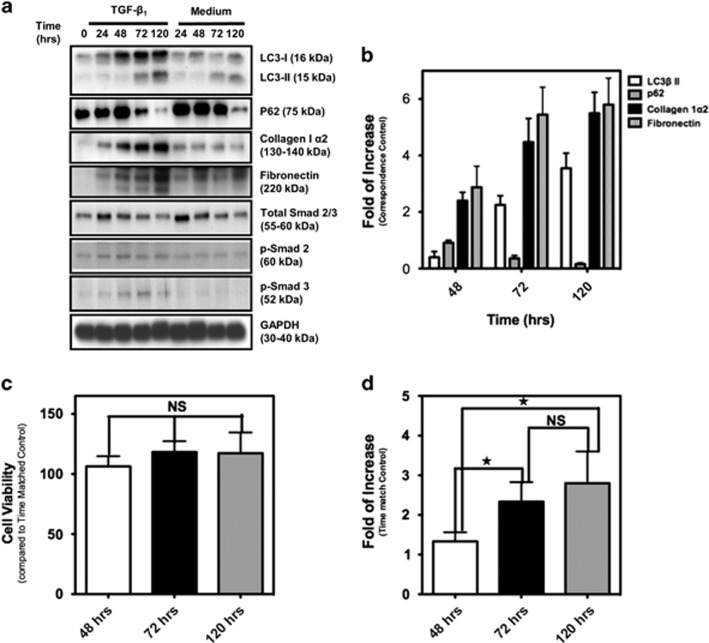

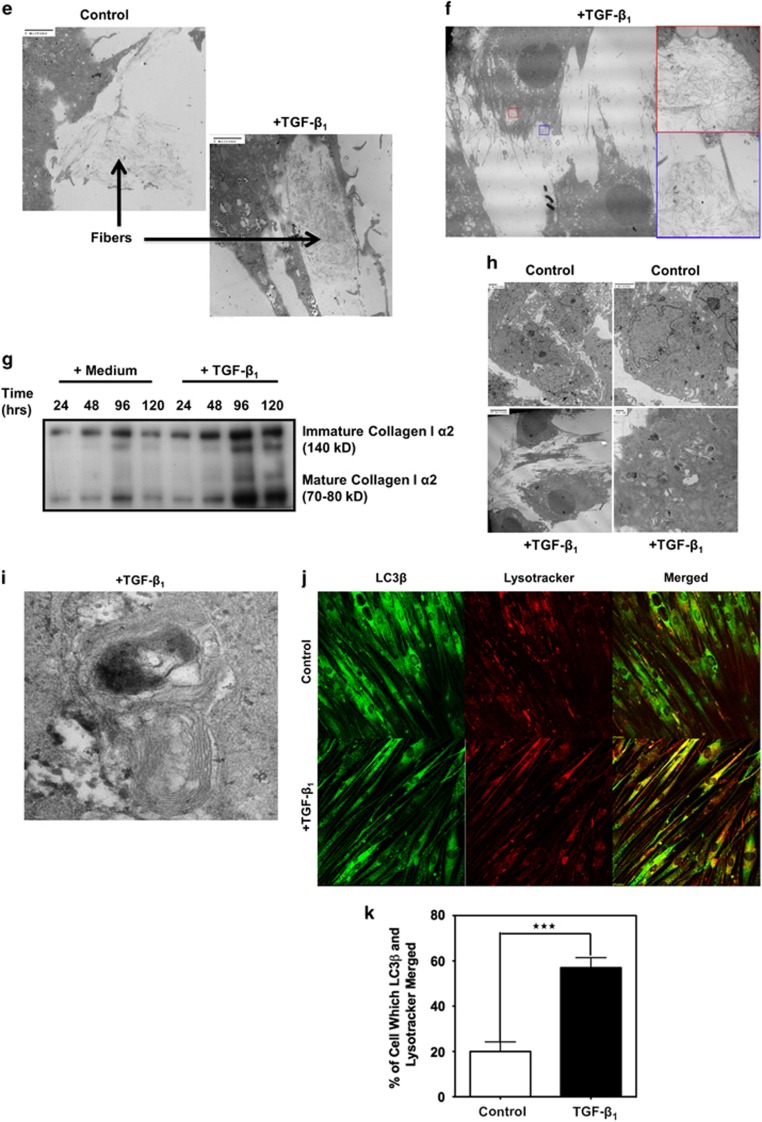
TGF*β*_1_ simultaneously induces fibrosis and autophagy in hATMyofbs. (**a**) Primary hATMyofbs (passages 2–5) were treated with TGF-*β*_1_ (10 ng/ml) for 0–120 h, and cell lysates were collected. Immunoblots were probed for the autophagy hallmark protein LC3*β* II, p62, as well as indicator proteins of the fibrogenic response in fibroblasts (i.e., collagen I*α*2, fibronectin and the Smad signaling pathway). TGF-*β*_1_ induced LC3*β* II lipidation, with parallel increases in collagen I*α*2, fibronectin protein expression and Smad2 and Smad3 phosphorylation. Data were normalized to GAPDH levels. Results are the means of three independent experiments from four different donors. (**b**) Densitometric analysis of LC3*β* II, p62, collagen I*α*2 and fibronectin levels in hATMyofbs. Data are the means of three independent experiments from three different donors. For each experiment, LC3*β* II, collagen I*α*2 and fibronectin levels were compared with those from time-matched controls and normalized to GAPDH levels. (**c** and **d**) TGF-*β*1 treatment does not affect cell viability of hATMyofbs, but it associated with their proliferation at 72 and 120 h. hATMyofbs were exposed to TGF-*β*_1_ (10 ng/ml) for the indicated time points (48, 72, 120 h), and cell viability and proliferation was measured as described in the Materials and Methods section in three different culture experiments (*n*=3). TGF-*β*_1_ treatment was not associated with any significant changes in cell viability (*P<0.01) (**c**) while it induced significant hATMyofb proliferation at 72 and 120 h compared with 48 h (*P*<0.01). (**e** and **f**) hATMyofbs were either untreated or treated with 10 ng/ml TGF-*β*_1_ for 96 h. Cells were then imaged by TEM at a magnification of 15 600 (**e**) and 6750 (**f**). Extracellular fiber deposition (collagen type I or fibronectin) was compared between the control and TGF-*β*_1_ treatment groups. TGF-*β* increased extracellular fiber deposition. (**g**) hATMyofbs were either untreated or treated with 10 ng/ml TGF-*β*_1_ for the indicated time points (0–120 h). The cell culture medium was collected and concentrated with filter tube (MESH 20 kDa). Collagen I*α*2 was probed in concentrated cell culture media. TGF-*β*1 increased mature and immature collagen secretion at different time points. (**h** and **i**) hATMyofbs were either untreated or treated with 10 ng/ml TGF-*β*_1_ for 96 h. Cells were then imaged by TEM at a magnification of 3600 (control, top panel left), 7500 (control, top panel right) and for TGF-*β*_1_ treatment (right panel 2750, left panel 27 500 and (**i**) 127 000). An autophagosome is highlighted in panel (**i**). (**j**) hATMyofbs treated with TGF-*β*_1_ (10 ng/ml, 96 h) showed increased LysoTracker Red DND-99 staining (a marker of lysosomal activation) and an increase in punctuate staining for LC3*β* (green), a marker of autophagy, and LC3*β* lysosomal co-localization. (**k**) hATMyofbs were treated with TGF-*β*_1_ (10 ng/ml, 96 h) and were immunostained for LC3*β* (green) and lysosomes (red). Ten different fields (10 cells in each field) were randomly chosen in control and TGF-*β*_1_ treatment and were counted manually by an operator. The percentage of yellow cells (merged LC3 and lysosomes) were compared between control and TGF-*β*_1_ treatment. TGF-*β*_1_ significantly increased the percentage of yellow cells, which indicated LC3*β* II lysosomal co-localization (***P <0.001). NS, not significant

**Figure 2:**
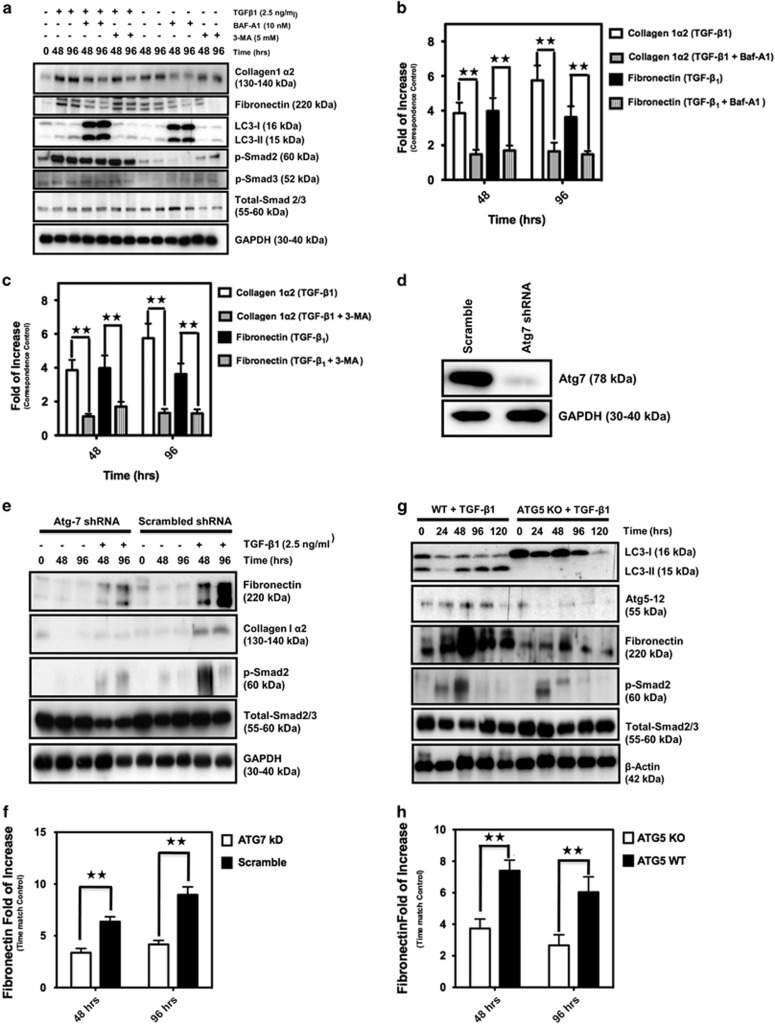

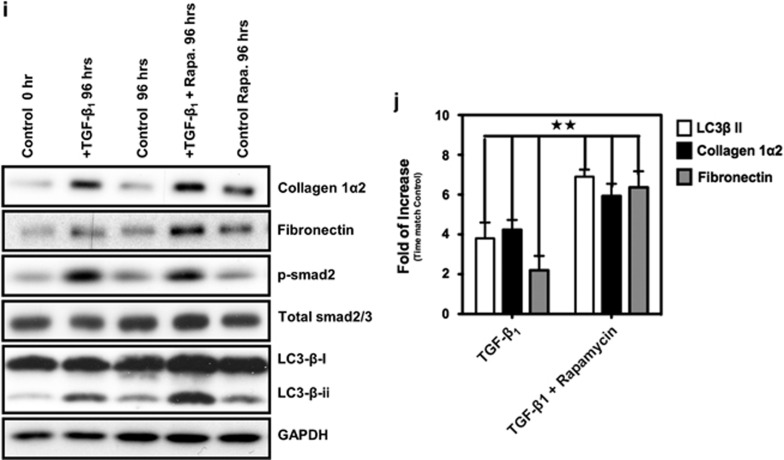
TGF-*β*_1_-induced autophagy is a requisite of TGF-*β*_1_-induced pro-fibrosis in hATMyofbs. (**a**) Primary hATMyofbs were treated with TGF-*β*_1_ (10 ng/ml) in the presence of the autophagy inhibitors Baf-A1 (10 nM) and 3-MA (2.5 mM) for the indicated durations. Western blotting analysis revealed that inhibition of autophagy abrogated the fibrogenic effects of TGF-*β*_1_ (i.e., decreased collagen type I*α*2 and fibronectin expression levels), whereas this treatment did not affect Smad2 or Smad3 phosphorylation. Equal protein loading was confirmed using GAPDH levels. Results are the means from three independent experiments using cells from three different donors. (**b** and **c**) Densitometric analysis of collagen I*α*2 and fibronectin levels in hATMyofbs, which were stimulated with TGF-*β*_1_ or autophagy inhibitors (i.e., Baf-A1 (10 nM) (**b**) or 3-MA (2.5 mM) (**c**). Inhibition of autophagy significantly decreased collagen I*α*2 and fibronection biosynthesis. Data are the means of three independent experiments using hATMyofbs from three different donors. For each experiment, collagen I*α*2 and fibronectin levels were compared with those from time-matched controls and normalized to GAPDH levels. (**d**–**f**) Protein required for autophagy induction (Atg7) were stably knocked down in hATMyofbs. Atg7 knocked down cells and their correspondence scramble infected cells were treated with TGF-*β*_1_ (10 ng/ml) for 48 and 96 h. Whole-cell lysates were extracted and then collagen I*α*_2_ and fibronectin expression levels were measured in the cell lysates. Protein loading was confirmed using GAPDH. (**f**) Densitometry analysis showed that Atg7 knockdown was associated with a significant (*P*<0.01) decrease of TGF-*β*_1_-induced fibronectin biosynthesis in hATMyofbs. (**g** and **h**) Atrg5 KD MEF were treated with TGF-*β*1 (10 ng/ml) for the indicated time points. LC3 lipidation, Atg5-12 conjugation fibronectin expression, total Smad 2/3 and Smad2 phosphorylation were measured in whole-cell lysates. Protein loading was confirmed using *β*-actin. (**g** and **h**) Densitometry analysis (**h**) revealed that *ATG5* knockdown was associated with a significant (*P*<0.01) decrease in TGF-*β*_1_-induced fibronectin biosynthesis in MEF. (**i** and **j**) Autophagy induction increases TGF-*β*_1_-induced fibrogenic effects. hATMyofbs were pretreated with Rapaymcin (4 h, 1000 nM) and then co-treated with TGF-*β*1 (10 ng/ml) for the indicated duration. LC3 lipidation, collagen type1 *α*2 expression, fibronectin expression, total Smad 2/3 and Smad2 phosphorylation were measured in whole-cell lysates. Protein loading was confirmed using GAPDH. (**j**) Densitometry analysis showed that Rapamycin (1000 nM) significantly (*P*<0.01) increased TGF-*β*_1_-induced fibronectin and collagen type 1 *α*2 biosynthesis in hATMyofbs. **P<0.01

TGF-*β*_1_ is but one of an array of factors shown to be involved in the induction of cardiac fibrosis, as demonstrated in overexpression and knockout models.^28, 29, 30^ As atrial fibrillation is a serious clinical problem with high incidence in society^31^ and is linked to fibrosis of atrial tissues,^32^ we investigated whether or not there was an association between TGF-*β*_1_-induced fibrosis and autophagy in hATMyofbs. Primary hATMyofbs constitute a clinically relevant model for the study of TGF-*β*_1_-induced fibrosis and autophagy. Our results show that TGF-*β*_1_ (10 ng/ml) induces significant increases in the synthesis of collagen type I*α*2 and fibronectin in the presence of LC3*β* II lipidation and increases Smad2 and Smad3 phosphorylation and p62 degradation (Figures 1a and b). We also showed that TGF-*β*_1_ treatment significantly did not affect the viability of hATMyofb cells (Figure 1c) while conversely it is associated with a significant induction of their proliferation at 72 and 120 h after treatments when compared with 48-h cultures (Figure 1d; *P*≤0.01). Fibrillar collagen type I and fibronectin deposition was also confirmed using transmission electron microscopic (TEM) images from hATMyofbs, which were stimulated with TGF-*β*_1_ at 10 ng/ml for 96 h (Figures 1e and f). Figures 1e and f indicate an increase of fiber deposition after TGF-*β*_1_ treatment. TGF-*β*_1_ stimulation increased collagen I*α*2 secretion from hATMyofb cells compared with time-matched control cells, which also proved TGF-*β*_1_-induced fibrosis (western blotting analysis in Figure 1g). Possible TGF-*β*_1_ autophagy induction was further investigated using TEM images from hATMyofbs stimulated with TGF-*β*_1_ at 10 ng/ml for 96 h. Figures 1h and i clearly show autophagosome and autophagolysosmes in hATMyofbs stimulated with TGF-*β*_1_. Moreover, our immunocytochemistry data indicate punctate LC3*β* II staining and lysosomal activation in hATMyofbs treated with TGF-*β*_1_ (Figure 1j), with a significant (*P*<0.001) increase in the number of cells. In this case, LC3*β* and lysotracker were co-localized (Figure 1k), which is consistent with the autophagy activation in these cells. To obtain more quantitative assessment of the induction of autophagy, we used bafilomycin-A1 (Baf-A1; 10 nM) to block the fusion of autophagosomes and lysosomes and assess the presence of autophagy flux.^13, 33, 34^ As shown in Figure 2a, we demonstrate autophagic flux, for example, autophagosome delivery to lysosomes and autophagolysosome formation, by co-treating hATMyofbs with TGF-*β*_1_ and Baf-A1 for 48 and 96 h. TGF-*β*_1_-induced accumulation of LC3-II was enhanced in the presence of Baf-A1, which supports the suggestion that TGF-*β*_1_ enhances autophagosome synthesis.

### TGF-*β*_1_-induced autophagy is required for fibrosis in hATMyofbs

To determine whether or not the link between autophagy and TGF-*β*_1_-induced fibrosis was more than correlational, we examined the effects of TGF-*β*_1_ stimulation of hATMyofbs in the presence of autophagy inhibitors. Baf-A1 and 3-methyladenine (3-MA) are known pharmacological inhibitors of autophagy, as shown by our group and others.^13, 33, 34, 35^ Thus we co-treated hATMyofbs with Baf-A1 (10 nM) or 3-MA (2.5 mM) and TGF-*β*_1_ (10 ng/ml) for 48 and 96 h, and collagen type I*α*2 and fibronectin expression levels were then compared with their corresponding controls (Figures 2a–c). We found that Baf-A1 or 3-MA co-treatment significantly inhibits TGF-*β*_1-_induced pro-fibrotic effects in hATMyofbs (Figures 2b and c; *P*<0.01).

Densitometric analysis of LC3*β*-II in cotreated myofibroblasts (TGF*β* and autophagy inhibitors) *versus* controls revealed that TGF-*β*_1_ treatment is associated with a 2.58±0.24-fold increase in LC3*β*-II at 48 h *versus* control and a 2.49±0.26-fold increase in LC3*β*-II at 96 h wherein the sample size is three. This equated to significance of *P*<0.01 when comparing to corresponding Baf-A1-treated preparations to time-matched controls. We suggest that decreased autophagy in the presence of autophagy inhibitors is linked to reduced basal production of matrix component proteins and thus that autophagy is positively correlated to the synthesis of matrix components and myofibroblast function.

To utilize a parallel non-pharmacological approach to test the same hypothesis, ATG7 gene expression was suppressed using stable lentiviral shRNA in hATMyofbs (Figure 2d) and later treated with TGF-*β*_1_. These cells were compared with hATMyofbs, which were infected with scrambled shRNA and collagen type I*α*2 and fibronectin expression levels were then compared. The results showed that ATG7 KD significantly (*P*<0.01) decreased TGF-*β*_1_-induced pro-fibrotic effects (Figures 2e and f).

We further used human embryo fibroblast (MEF) cells wild type (WT) and *ATG5* knockout (*ATG*5 KO) and showed that TGF-*β*_1_ stimulation induced significantly (*P*<0.01) less fibronectin synthesis in MEF *ATG5* KO cells compared with corresponding WT cells (Figures 2g and h). We also induced autophagy in hATMyofbs using Rapamycin (in a concentration of 1000 nM for a duration of 96 h) and investigated TGF-*β*_1_-induced fibrogenic effects. Our experiment showed that autophagy induction significantly (*P*<0.01) increased the TGF-*β*_1_-induced fibrogenic effect (Figures 2I and j).

### Coincidence of elevated autophagy markers and fibronectin induction in scar tissue from post-MI rats

**Figure 3:**
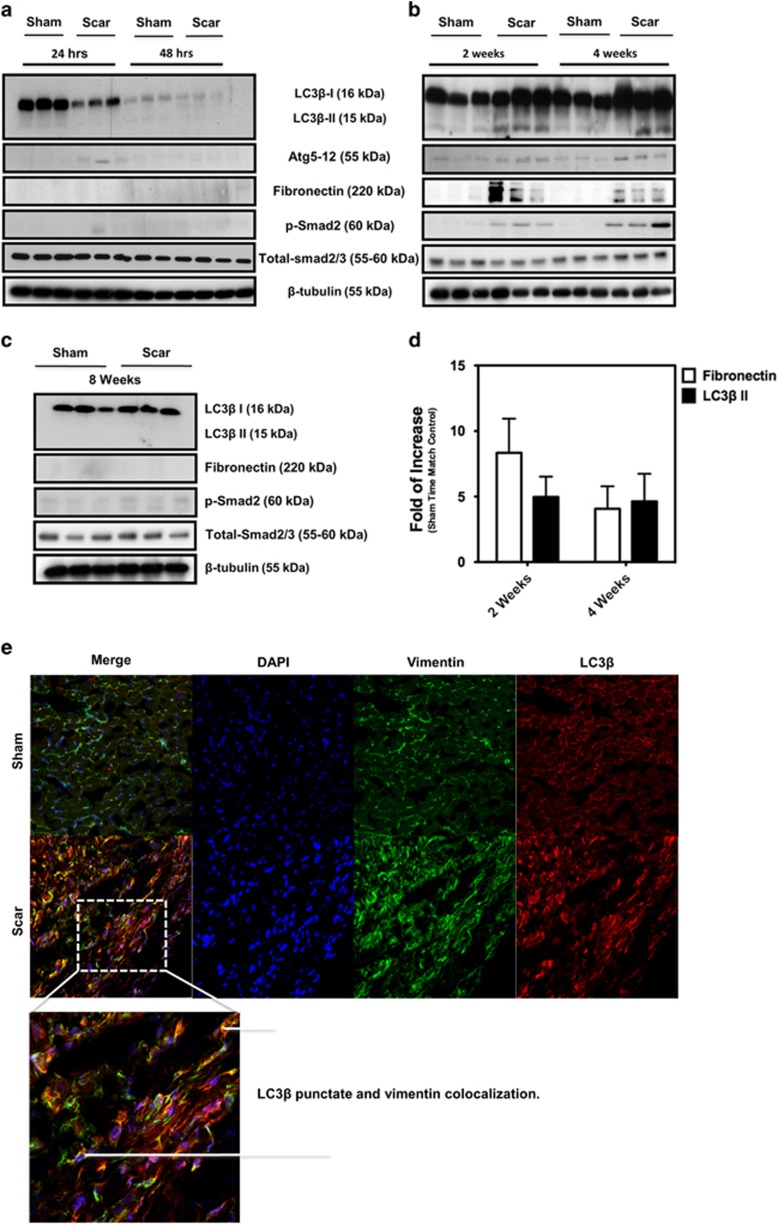

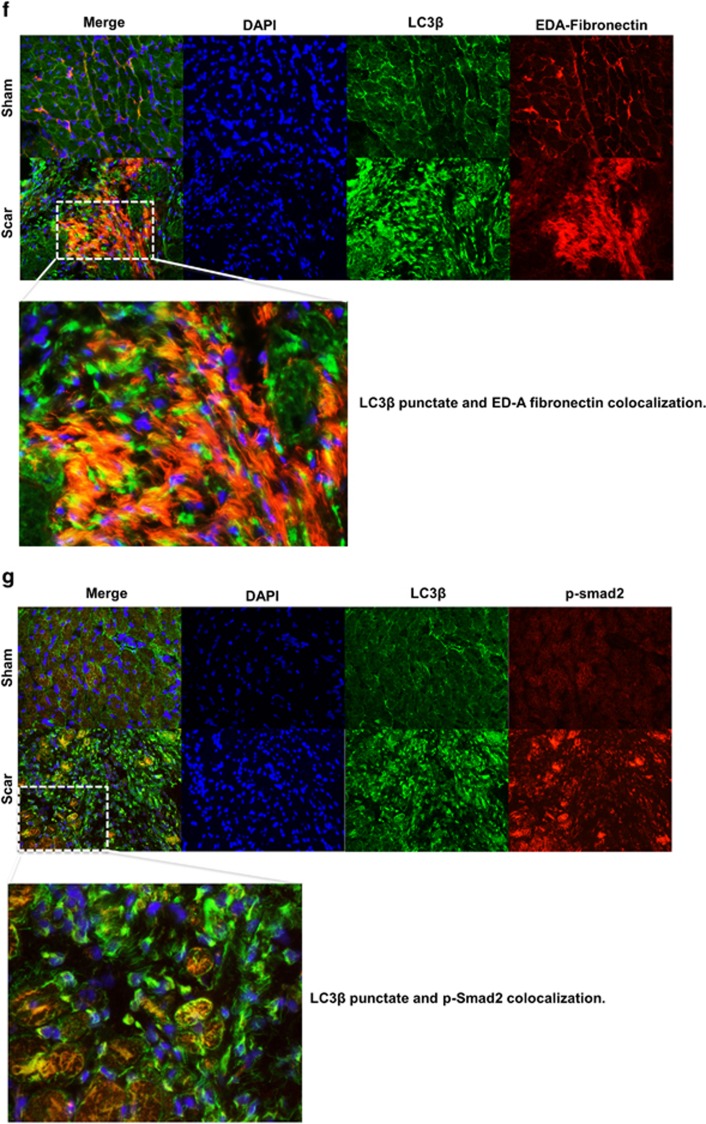
Concomitant occurrence of autophagy, fibrosis and Smad2 phosphorylation in scar tissue from post-MI rats. (**a**–**c**) Western blotting analysis of infarct scar and non-infarcted (NI) control tissues from post-MI experimental animals confirmed the concomitant occurrence of fibrosis (i.e., elevation of fibronectin levels), autophagy (i.e., elevation of LC3*β* II and Atg5-12 levels) and Smad2 phosphorylation, with Smad phosphorylation (p-Smad) being a hallmark of TGF-*β*_1_ activation, in scar tissue 2 weeks after MI. This time point was chosen as it reflects the active healing phase after MI. (**d**) Densitometry analysis of fibronectin and LC3*β*-II of tissues from post-MI experimental animals showed that both proteins have increased in scar area compared with sham area at 2 and 4 weeks time point. (**e**–**g**) Immunofluorescence histochemical analysis of sham and scar tissue showed the co-localization of LC3*β* with vimentin (**e**), EDA-Fibronectin (**f**) and phospho Smad2 (**g**) in scar compared with sham area

MI is a major cause of congestive heart failure,^36, 37, 38^ and increased levels of TGF-*β*_1_ mRNA and protein expression are evident in the myocardium bordering the infarct region 2 days following MI.^39^ This observation is highly suggestive of a crucial role for TGF-*β*_1_ in cardiac wound healing and the fibrotic response. Thus, we investigated Smad2 phosphorylation, as well as the levels of autophagy and fibrosis markers, on scar and sham-operated tissues from our rat model of MI. As shown in Figure 3b, Smad2 phosphorylation, autophagy markers (e.g., LC3*β* II lipidation and Atg5-12 conjugate) and fibronectin synthesis are all increased in scar tissue at 2 and 4 weeks post-MI (Figures 3b and d). These findings positively correlate with the elevated expression levels of dimeric TGF-*β*_1_ (e.g., the 25-kDa band) that we have observed at 2 and 4 weeks post-MI *versus* non-infarcted control and sham-operated left ventricular muscle.^40^ However, we found no evidence of Smad2 phosphorylation or changes in the levels of autophagy markers and fibronectin synthesis at 24 or 48 h or at 8 weeks post-MI (Figures 3a and c). We also showed that LC3*β* punctate staining co-localized with vimentin (a mesenchymal marker – see Figure 3e), ED-A fibronectin (Figure 3f) and phosphorylated Smad2 (Figure 3g – wherein Smad2 phosphorylation is a marker of canonical TGF-*β*_1_ activation), which supports our hypothesis about the role of autophagy and TGF-*β*_1_ in fibrosis induction in MI tissue.

## Discussion

In this study, we have shown that TGF-*β*_1_ simultaneously induces autophagy and fibrosis in human atrial myofibroblasts and that pharmacological inhibition of autophagy is associated with a parallel reduction in TGF-*β*_1_-induced fibrosis. Our findings highlight a linkage between autophagy and elevated matrix protein synthesis by hATMyofbs and TGF-*β*1 activation in scar tissue from a rat model of MI. These results strongly support the hypothesis that TGF-*β*_1_-induced fibrosis depends upon its ability to induce autophagy.

Basal level autophagy occurs in all cell types but can be rapidly upregulated as an adaptive response to generate intracellular nutrients and energy under conditions of cellular stress.^41, 42^ Autophagy is a tightly regulated process that is a highly conserved, phylogenetically ancient process. It is observed in yeast cells to mammalian tissues and has roles in various biological events, such as cellular remodeling during development, differentiation, adaptation to changing environmental conditions, lifespan extension and response to environmental stress.^11, 43, 44^ In contrast, uncontrolled autophagy can drive cells towards type II programmed cell death, which is morphologically distinct from type I (e.g., apoptosis), and may be involved in the pathogenesis of a number of different diseases.^44, 45, 46, 47^ This notwithstanding, a growing body of evidence points to autophagy as being important for cell survival and that an accumulation of autophagosomes may simply reflect a survival response to deadly stress aimed at ridding the cell of misfolded proteins or damaged organelles.^13, 33, 48^ Although cellular necrosis is generally accepted as the major mechanism for cell death in post-MI cardiac tissues, we note several recent reports that address the occurrence of autophagy in post-MI myocardium. Kanamori *et al.*^49^ have reported that autophagy is activated in cardiomyocytes and specifically that autophagic activity was particularly strong in salvaged cardiomyocytes bordering the infarcted area. Kanamori *et al.*^50^ have reported that during both subacute and chronic post-MI stages, for example, at 1 week and 3 weeks after MI, respectively, autophagy is activated in surviving cardiomyocytes, as they have demonstrated by the upregulated expression of microtubule-associated protein-1 light chain 3-II (LC3-II), p62 and cathepsin D and by electron microscopic findings. Their finding closely correlate with our findings in the rat experimental model of infarction that we have currently used (Figure 3b). Whelan *et al.*^51^ have also recently suggested a possible role for autophagy in post-MI tissues, and thus the argument that autophagy occurs in and may regulate cellular responses in post-MI heart in addition to necrosis is established in the literature. With regard to the question of whether autophagy could be a back-up mechanism for fibrosis – there are several hypotheses that we are testing in our current research program. One of these mechanisms addresses the role of autophagy in providing energy for pro-fibrotic protein biosynthesis.

The ECM provides a scaffold that surrounds and supports cells in virtually all tissues.^52^ Tissue fibrosis is the structural basis for a variety of chronic human diseases, including cardiovascular fibrosis,^53^ liver cirrhosis, end-stage kidney disease, systemic sclerosis and various autoimmune diseases,^54^ and causes irreversible damage to affected tissues, including inotropic and lusitropic dysfunction in the heart. Despite recent progress in understanding the mechanisms underlying the pathogenesis of tissue fibrosis, and developing novel therapeutic strategies to reverse it,^55, 56^ there are no effective therapeutic treatments presently available to combat fibro-proliferative diseases.^57^

TGF-*β*_1_ induces both autophagy^24, 58^ and fibrosis in many tissues,^27, 59^ and the concomitant occurrence of autophagy and fibrosis in many diseases has been previously observed.^27, 60, 61^ We used pharmacological inhibitors of autophagy, including the class III phosphoinositol 3-kinase inhibitor, 3-MA^13, 60^ and the lysosomal ATPase pump inhibitor, Baf-A1,^33, 61^ to show that inhibition of autophagy significantly attenuates TGF-*β*_1_-mediated pro-fibrotic effects in hATMyofbs. Our results are supported by studies demonstrating that autophagy is necessary for induction of fibrosis in hepatic cells.^27, 62^

We extended our *in vitro* observations using hATMyofbs by including an *in vivo* model of MI in rats. Cardiac fibroblasts are key players in maintaining homeostasis of the heart's ECM.^53^ Cardiac ECM remodeling is well documented in post-MI hearts in the infarct zone, as well as in both ventricles remote to the infarct scar. In other cardiac diseases (such as hypertension), globalized fibrosis is likely a primary contributor to the progression of congestive heart failure.^63^ It is well known that TGF-*β*_1_ is expressed in the heart^64^ and that, following MI, the levels of TGF-*β*_1_ are increased in the scar area.^7^ Here, we show that indicators of autophagic induction (i.e., LC3*β* II, Atg5-12 conjugation, fibronectin synthesis and Smad2 phosphorylation) are increased in lysates of cardiac scar tissue at 2 and 4 weeks post-MI, as compared with sham-operated heart tissues. These findings correlate with the temporal window for infarct scar healing in post-MI rat heart following coronary arterial occlusion.^65^ Conversely, samples taken at 24 and 48 h, and at 8 weeks post-MI, show no signs of autophagic activation in terms of LC3*β* II, Atg5-12 conjugation, fibronectin synthesis or Smad2 phosphorylation, in either scar or sham-operated control tissue. Together, these data establish the co-incidence of autophagy and fibrosis in the presence of increased TGF-*β*_1_ levels in a rat model of MI and directly correlate with our *in vitro*, cell-based studies with hATMyofbs. More generally, as blockade of autophagy in fibrogenic cells from different organs also attenuates fibrogenesis, it appears that autophagy is an evolutionarily conserved, core pathway that contributes to, and regulates, the fibrotic response in a wide range of tissues.^66^

Our findings underscore the parallel roles played by autophagy and fibrosis in the processes involved in cellular activation and enhanced ECM production in the heart. They also provide a novel framework for understanding the basis of cardiac fibrotic disease, as well as the regulation of fibrosis in general. Finally, these results highlight autophagy as a putative novel therapeutic target in attenuating fibrosis in fibro-proliferative diseases.

## Materials and Methods

### Reagents

Cell culture media and supplements (i.e., SMEM, DMEM, fetal bovine serum (FBS), penicillin/streptomycin (Pen/Strep) and insulin/transferrin/selenium (ITS)) were obtained from Gibco (Thermo Fisher Scientific Inc., Waltham, MA, USA), and collagenase I type *α*2 was from Santa Cruz Biotechnology (Santa Cruz, CA, USA) (sc-8786). Phenylmethanesulfonyl fluoride (PMSF), protease inhibitor cocktail, *β*-glycerol 3-phosphate and simvastatin were obtained from Sigma-Aldrich (St. Louis, MO, USA), and enhanced chemiluminescence (ECL) reagents were purchased from Amersham-Pharmacia Biotech Inc. (Piscataway, NJ, USA). Baf-A1 and 3-MA were from Sigma-Aldrich, and LysoTracker Red DND-99 was obtained from Molecular Probes (Eugene, OR, USA). All other reagents were of ACS Grade or better. Human ATG7 shRNA and human scrambled shRNAs were purchased from Santa Cruz Biotechnology.

### Antibodies

Rabbit anti-human/mouse/rat LC3*β* II was from Sigma-Aldrich. Rabbit anti-human Atg5, Smad2/3, phospho-Smad2, phospho-Smad3, Atg7 and p62 antibodies were purchased from Cell Signaling (Danvers, MA, USA). Rabbit anti-human fibronectin, mouse anti-human glyceraldehyde-3-phosphate dehydrogenase (GAPDH) and goat anti-human collagen 1*α*2 were obtained from Santa Cruz Biotechnology. Horseradish peroxidase- and fluorochrome-conjugated secondary antibodies were purchased from Sigma-Aldrich.

### Primary hATMyofb cell culture preparation

Approval was obtained from the Research Ethics Board of the University of Manitoba for the collection of atrial tissue from patients undergoing cardiac surgical procedures. Written, informed consent was obtained from each patient prior to tissue collection. We declined samples from patients with atrial fibrillation, and our patient donors are typically preinfarct coronary artery bypass graft (CABG) candidates. Furthermore first- and second-passage human atrial fibroblasts (P1 and P2) from this patient group show a high degree of similarity among preparations from different individual patients, as simple plating of these cells in two dimensions on plastic plates has a greater impact on cellular phenotype than any other factor. These conditions tend to provide equalization of phenotype prior to experimentation and thus allow meaningful comparisons between cells harvested from different patients.

We specifically used medium containing ITS in all of the cell culture experiments included herein. We felt that this is a superior culture system to medium containing FBS, as FBS contains unknown factors that may confound the interpretation of the results of these experiments. Conversely, the ITS medium contains factors (detailed above) to prevent extreme autophagy induction.

Fragments of atrial tissue were subjected to collagenase digestion to isolate cardiac myofibroblasts. Thus the primary human atrial myofibroblasts used in these studies are cells isolated directly from the atrial appendage during various cardiac surgical procedures. Minced atrial tissue was treated with 2 mg/ml collagenase in SMEM and incubated for 3 h at 37 °C in an atmosphere of 95% O_2_ and 5% CO_2_. Therefore atrial tissue is dissociated with collagenase and the dissociated cells of interest (cardiac fibroblasts) adhere rapidly to cell culture plates, for example, within 3 h of plating. Other cells are washed away after initial plating, including relatively non-adherent myocytes, epithelial cells and others. Collagenase was neutralized by the addition of an equal volume of DMEM/F12+20% FBS, and liberated cells were collected by centrifugation at 800 × *g* for 7 min at room temperature. Cells were re-suspended in fresh DMEM/F12+20% FBS, seeded onto plastic culture dishes and incubated overnight at 37 °C in 95% O_2_ and 5% CO_2_. The following day, fresh DMEM/F12+20% FBS was added to the adherent cells, and then media was replaced every 3 or 4 days thereafter.^33^ Cells are cultured until nearly confluent and then passaged a maximum of two times (P2), which yields a relatively pure population of cardiac myofibroblasts.

Myofibroblasts are well known to arise from a range of cellular precursor sources. *In vivo*, these may include resident fibroblasts, pericytes, fibrocytes, bone marrow stem cells, epithelial-to-mesenchymal transition and endothelial-to-mesenchymal transition. Once these cells become established in the infarct scar, cardiac myofibroblasts persist within the scar for months and years following initial injury.^67^ Primary fibroblasts (and myofibroblasts) adhere more readily and quickly to culture plates than other cell types in the heart resulting in a very pure population of fibroblasts/myofibroblasts during isolation procedures. However, once in culture, fibroblasts readily differentiate (<16 h) into myofibroblasts. Thus in using these cells, we can be assured that the cells are myofibroblasts, and we have previously published the characteristics of these cells during different passages.^68^ In addition, the cells are isolated from the atrial appendage of diseased human hearts, which already have a substantial population of myofibroblasts.

### Cell viability assay

Human atrial myofibroblasts (passage 1 or 2) were cultured in 96-well plates (20 000 cells per well). After reaching a confluency of around 60%, they were treated with TGF-*β*1 (10 ng/ml) for different time points (48, 72, 120). At each time point, the cell viability was assessed using MTT [(3-(4,5-dimethylthiazol-2-yl)-2,5-diphenyltetrazolium bromide) assay as we have described before.^69^

### Cell proliferation assay

Human atrial myofibroblasts (passage 1 or 2) were cultured in 96-well plates (20 000 cells per well). After reaching a confluency of around 60%, they were treated with TGF-*β*1 (10 ng/ml) for different time points (48, 72, 120). At each time point, the cell numbers was measured using cell counting in the presence of Trypan blue as we have described before.^70^

### Immunohistochemistry

Tissue was prepared according to procedures as described^71^ with the exception of tissue fixation in 95% ethanol. Briefly, hearts were excised from animals killed at 2 weeks after left ventricular coronary ligation surgery. Isolated left ventricular tissue was frozen in embedding medium at −80 °C to be used for sectioning. Frozen tissue blocks were cut in 7-*μ*M thick transverse sections across the ventricular scar area using a Microm HM 550 cryotome and placed on slides. Tissue was then fixed in 4% paraformaldehyde for 15 min washed 3 × in 1% PBS buffer and permeabilized with 0.1% triton X-100 for 15 min. Tissue slices were washed 3 × 15 min in 1% PBS and incubated overnight at 4 °C with primary antibodies (Vimentin 1 : 200, LC3 1 : 200, ED-A fibronectin 1 : 100 and p-Smad2 1 : 20) in 1% bovine serum albumin. Tissue slices were washed the next day 3 × for 15 min with 1% PBS and incubated for 90 min at room temperature with fluorescently conjugated secondary antibodies. Slides were washed again 3 × for 15 min with 1% PBS and then dried. Cover slips were then mounted onto slides using Prolong Gold antifade reagent (Thermo Fisher Scientific Inc.) with DAPI. Images were visualized and captured using an epifluorescence equipped microscope (Leica Microsystems TCS SP5 Confocal Microscope, Buffalo Grove, IL, USA) at × 40 magnification.

### Stable gene silencing: lentiviral delivery of shRNA

Human atrial myofibroblasts (passage 1) were infected by *ATG7* shRNA- and noncoding shRNA-lentiviral particles (Santa Cruz Biotechnology), and stable clones were selected using puromycin.^33, 34^

### Stimulation of hATMyofbs with TGF-*β*_1_

Primary human atrial myofibroblasts (passage 1 or 2), hATMyofb *ATG7* KD, hATMyofbs infected with noncoding shRNA, MEF (WT), and *ATG5* KO MEF were grown in 100-mm cell culture dishes up to 80% confluency in DMEM+10% FBS. In preparation for experimentation, cells were starved in DMEM+1% ITS for 48 h,and then stimulated with TGF-*β*_1_ (10 ng/ml) in DMEM+1% ITS for 0–120 h. In experiments using Baf-A1, 3-MA and Rapamycin, the cells were first pretreated with Baf-A1 (10 nM), 3-MA (2.5 mM), Rapamycin (1000 nM) for 4 h prior to the addition of TGF-*β*_1_ (10 ng/ml) for various time points thereafter.

### Secreted collagen I*α*2 assay

Collagen I*α*2 secretion was assessed by immunoblot analysis. Primary human atrial myofibroblasts (passages 2–5) were seeded in 100-mm dishes. Cells were treated with TGF-*β*_1_ for the indicated time points (0–120 h), the supernatant was collected centrifuged in 10 000 × *g* for 20 min, transferred to 50-ml filter tubes (20-kD mesh, Amicon, Millipore, Bedford, MA, USA) and centrifuged at 3000 × *g* for 35 min, The remaining supernatant on top of the filter was collected, and western blotting analysis was performed using a goat anti- Collagen I*α*2 antibody (Santa Cruz Biotechnology, USA).

### Immunoblotting

Western blotting analysis was used to detect LC3*β* II, Atg5-12, Smad2/3, phospho-Smad2, phospho-Smad3, collagen type 1*α*2, fibronectin, p62, Atg7, Atg5 and GAPDH as previously described.^13^ Briefly, cells were washed with Tris-buffered saline (TBS), and protein extracts were prepared in lysis buffer (20 mM Tris-HCl (pH 7.5), 0.5% Nonidet P-40, 0.5 mM PMSF, 100 *μ*M *β*-glycerol 3-phosphate and 0.5% protease inhibitor cocktail). Following centrifugation at 13 000 × *g* for 10 min, supernatant protein content was assayed according to the bicinchoninic acid method. Proteins were size-fractionated by SDS-PAGE under reducing conditions and subsequently transferred to PVDF membranes. Blots were blocked with 10% non-fat dried milk in TBS+0.02% Tween 20 and then incubated overnight with primary antibodies in 5% non-fat dried milk in TBS+0.02% Tween 20 at 4 °C. Blots were subsequently incubated with HRP-conjugated secondary antibodies for 1 h at RT in 3% non-fat dried milk in TBS+0.02% Tween 20, and proteins were visualized with ECL on X-ray film.

### Immunocytochemistry, confocal imaging and TEM

In immunocytochemistry studies, hATMyofbs were grown overnight on Tcoverslips and then treated with simvastatin (10 *μ*M) or vehicle for 72 h. Lysosomes were stained with LysoTracker Red DND-99 (100 nM × 10 min) prior to fixation in 4% paraformaldehyde/120 mM sucrose and permeabilization with 3% Triton X-100. Cells were then incubated with rabbit anti-LC3*β* II IgG (1 : 200) with a corresponding fluorochrome-conjugated secondary antibody. Fluorescence images were captured and analyzed using an Olympus FluoView multi-laser confocal microscope (Olympus Canada Inc., Toronto, ON, Canada). For TEM, cells were fixed with 2.5% glutaraldehyde in PBS (pH 7.4) for 1 h at 4 ° and postfixed with 1% osmium tetroxide prior to embedding in Epon. TEM was performed using a Philips CM10 (Koninklijke Philips N.V. (Philips), Amsterdam, Netherland) at 80 kV on ultra-thin sections (100 nm on 200 mesh grids) stained with uranyl acetate and counter-stained with lead citrate as previously described.^13, 34^

### Experimental rat model of MI

Experimental protocols for animal studies were approved by the Animal Care Committee of the University of Manitoba, Canada and conformed to the guidelines established by the Canadian Institutes of Health Research and the Canadian Council on Animal Care (2001). MI was induced in male Sprague-Dawley rats (150–175 g) by surgical occlusion of the left coronary artery, as described previously.^65^ The mortality of the animals operated on in this manner was 30% within 48 h. Experimental animals were killed after 24, 48 h (e.g., acutely infarcted myocardium), as well as 2, 4 and 8 weeks (e.g., chronically infarcted) post-MI hearts, and cardiac tissues isolated from two left ventricular (HRP) regions, including remnant/viable (e.g., non-infarcted (NI)) LV free wall remote from the infarct scar and septum), and the infarct scar itself. Infarcted (e.g., pale, necrotic tissue in acutely infarcted *versus* overtly healed scar tissue in chronically infarcted animals) and non-infarcted (NI) regions were determined visually and compared with the same regions obtained from sham-operated rats. Samples were frozen in liquid N_2_ for subsequent western blotting analysis.

### Statistical analysis

Results are expressed as means±S.E.M., and statistical differences were determined by one- or two-way ANOVA, followed by Tukey's or Bonferroni's *post hoc* test using the Graph Pad Prism 5.0 software (GraphPad Software, Inc., La Jolla, CA, USA). A *P*-value of <0.05 was considered significant. Data were collected in triplicate from at least three separate cell cultures.

## Abbreviations

3 MA: 3-methyl
AF: atrial fibrillation
ANOVA: analysis of variance
ATG: autophagy related protein
Baf-A1: bafilomycin –A1
BSA: bovine serum albumin
CABG: coronary artery bypass graft
DAPI: 4',6-diamidino-2-phenylindole
DMEM: Dulbecco's modified Eagle's medium
ECL: enhanced chemiluminescence
ECM: extracellular matrix
ED-A fibronectin: extracellular domain - A fibronectin
FBS: fetal bovine serum
GAPDH: glyceraldehyde 3-phosphate dehydrogenase
hATMyofbs: human atrial myofibroblasts
HRP: horseradish peroxidase
ITS: insulin, transferrin, selenium augmented medium
LC3: microtubule-associated protein 1A/1B-light chain 3
LV: left ventricle
MEF: mouse embryonic fibroblast
MI: myocardial infarction
MTT: 3-(4,5-Dimethylthiazol-2-yl)-2,5-diphenyltetrazolium bromide
NI: non-infarcted
P1: first passage cell culture
PMSF: phenylmethylsulfonyl fluoride
SDS-PAGE: sodium dodecyl sulphate polyacrylamide gel electrophoresis
SEM: standard error of the mean
shRNA: short hairpin RNA
Smad2: Sma/Mad mothers against dipentaplegic 2
SMEM: Spinner modified essential medium
TBS: tris-buffered saline
TEM: transmission electron microscopy
TGF-b1: transforming growth factor beta 1
